# ChemSAR: an online pipelining platform for molecular SAR modeling

**DOI:** 10.1186/s13321-017-0215-1

**Published:** 2017-05-04

**Authors:** Jie Dong, Zhi-Jiang Yao, Min-Feng Zhu, Ning-Ning Wang, Ben Lu, Alex F. Chen, Ai-Ping Lu, Hongyu Miao, Wen-Bin Zeng, Dong-Sheng Cao

**Affiliations:** 1 Xiangya School of Pharmaceutical Sciences, Central South University, No. 172, Tongzipo Road, Yuelu District, Changsha, People’s Republic of China; 2 The Third Xiangya Hospital, Central South University, Changsha, People’s Republic of China; 3 Institute for Advancing Translational Medicine in Bone and Joint Diseases, School of Chinese Medicine, Hong Kong Baptist University, Kowloon Tong, Hong Kong SAR People’s Republic of China; 4 Department of Biostatistics, School of Public Health, University of Texas Health Science Center, Houston, TX 77030 USA

**Keywords:** Online modeling, Molecular descriptors, Machine learning, QSAR/SAR, Cheminformatics

## Abstract

**Background:**

In recent years, predictive models based on machine learning techniques have proven to be feasible and effective in drug discovery. However, to develop such a model, researchers usually have to combine multiple tools and undergo several different steps (e.g., *RDKit* or ChemoPy package for molecular descriptor calculation, *ChemAxon Standardizer* for structure preprocessing, *scikit*-*learn* package for model building, and *ggplot2 package* for statistical analysis and visualization, etc.). In addition, it may require strong programming skills to accomplish these jobs, which poses severe challenges for users without advanced training in computer programming. Therefore, an online pipelining platform that integrates a number of selected tools is a valuable and efficient solution that can meet the needs of related researchers.

**Results:**

This work presents a web-based pipelining platform, called ChemSAR, for generating SAR classification models of small molecules. The capabilities of ChemSAR include the validation and standardization of chemical structure representation, the computation of 783 1D/2D molecular descriptors and ten types of widely-used fingerprints for small molecules, the filtering methods for feature selection, the generation of predictive models via a step-by-step job submission process, model interpretation in terms of feature importance and tree visualization, as well as a helpful report generation system. The results can be visualized as high-quality plots and downloaded as local files.

**Conclusion:**

ChemSAR provides an integrated web-based platform for generating SAR classification models that will benefit cheminformatics and other biomedical users. It is freely available at: http://chemsar.scbdd.com.

**Graphical abstract:**
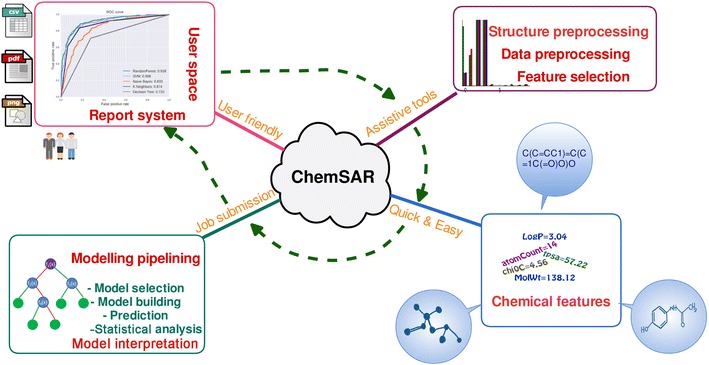
.

**Electronic supplementary material:**

The online version of this article (doi:10.1186/s13321-017-0215-1) contains supplementary material, which is available to authorized users.

## Background

The increasing availability of data on characteristics and functions of biomolecules and small chemical compounds enables researchers to better understand various chemical, physical and biological processes or activities with the use of machine learning methods [1–7]. Particularly in the drug discovery field, machine learning methods are frequently applied to build *in silico* predictive models in studies of structure–activity relationships (SAR) and structure–property relationships (SPR) to assess or predict various drug activities [8, 9], and ADME/T properties [10–16]. Nowadays, with the development of various public data sources (e.g., *ChEMBL* [17], *PubChem* [18], and *DrugBank* [19]), more and more scientific studies are utilizing predictive SAR/SPR models to perform virtual screening [20], study drug side effects [21–24], predict drug–drug interactions [25] or drug–target interactions [26–28], and investigate drug repositioning [29, 30]. Undoubtedly, robust and predictive SAR models built upon machine learning techniques provide a powerful and effective way for pharmaceutical scientists to tackle the aforementioned problems; however, there still exist two major barriers to overcome.

First, owing to the fusion of different scientific disciplines, a higher level of background knowledge and professional skills is required to solve many existing biological problems. For example, to reliably predict drug ADME/T properties, a researcher must be familiar with both pharmacokinetics and a modern programming language [14]; however, in many cases, researchers from the pharmaceutical or biomedical fields may lack formal training in computing skills. It may thus become necessary to save these investigators from tedious programming or deployment work such that they can focus on solving scientific problems.

Second, even if a researcher acquired the related background knowledge and computing skills, it is very time-consuming to build a predictive model as a number of steps are needed, including molecule representation, feature filtering, selection of a suitable machine learning method, prediction of new molecules, and relevant statistical analysis. In particular, the researcher needs to select and combine different tools to accomplish these steps; for example, using *RDKit* [31] to calculate molecular descriptors, using *libSVM* [32] or *scikit*-*learn* [33] to establish a model, using *ggplot2* [34] to plot or visualize the results. However, the selection and integration of such tools involve lots of programming issues and efforts.

For molecular representation, tools like *RDKit*, *CDK* [35], *Chemopy* [36], *OpenBabel* [37], *PaDEL* [38], Cinfony [39], PyDPI [40], Rcpi [41], have been developed to provide thousands of molecular descriptors. For building SAR/SPR models based on machine learning algorithms, a series of package have been implemented, including *scikit*-*learn* in Python, and *pls* [42], *earth*, *caret* [43], *randomFroest* [44], *kernlab* [45], and *RRegrs* [46] in R. Visualization packages like *matplotlib* [47], *ggplot2* and *seaborn* [48] are also freely available to produce high-quality statistical graphics. In addition, several online web services such as *ChemDes* [49], *BioTriangle* [50], *E*-*DRAGON* [51], *QSAR4U* [52] and *OpenTox* [53] are also available for drug discovery purpose. However, these tools are developed independently using different programming languages and APIs such that a unified and comprehensive platform is desirable to release biomedical investigators from such tedious and repeated efforts.

Toward this goal, a few previous studies made attempts to integrate one or two of the steps into a single package. For instance, the VCCLAB group [51, 54] developed E-DRAGON, an online platform for DRAGON software, to calculate various molecular descriptors, and also provided several online machine learning tools like PLSR and ASNN for model building. However, these tools are independent of each other and cannot be further integrated together to accomplish the entire modeling process. The *OCHEM* platform [55] was developed to provide a practical online chemical database. Also, this platform provides a modelling environment that enables users to standardize molecules, calculate molecular descriptors and build QSAR models. However, the *OCHEM* database is not specialized for SAR modelling and thus still lacks some essential functionalities like feature selection and advanced statistical analysis. Its modeling function is solely based on small molecules and thus cannot be used to analyze other independent biomedical dataset. More recently, Murrell et al. [56] developed a relatively well-integrated R package, named *camb*, for QSAR modeling. However, it does require users to have sufficient programming skills in R. In 2010, Chembench [57, 58] was developed and make progress in the simplicity of the use of QSAR modelling for analyzing experimental chemical structure–activity data. Other software applications that should be mentioned include *eTOXlab* [59], *AZOrange* [60], *QSARINS* [61], *OECD QSAR Toolbox* [62], *BuildQSAR* [63], *Molecular Operating Environment* (*MOE*) [64] and *Discovery Studio* (*DS*) [65]; however, these software are either commercial or difficult for users to deploy by themselves.

**Fig. 1:**
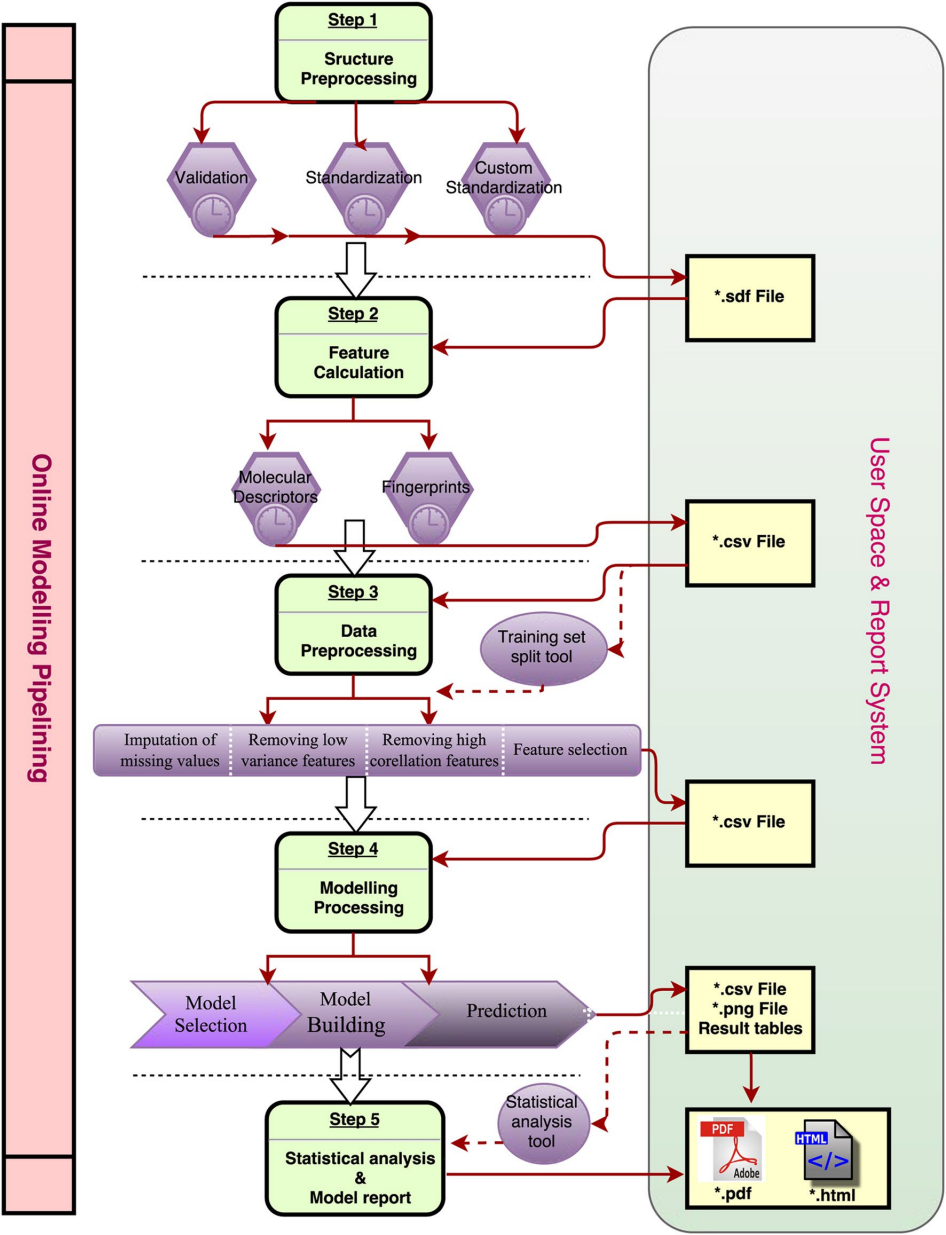
The pipelining of ChemSAR. It contains six main modules: (1) *User space*, (2) *Structure preprocessing*, (3) *Data preprocessing*, (4) *Modelling process*, (5) *Model interpretation* (6) *Tools*. Each of them not only could be utilized as one part of the whole pipelining but also could be used as an independent tool

In view of these limitations, we implemented a web-based platform, called ChemSAR, as an online pipelining for SAR model development. ChemSAR integrates a set of carefully selected tools and provides a user-friendly web interface and allows users to complete the entire workflow via a step-by-step submission process without involving any programming effort. Currently, ChemSAR is mainly designed for molecular SAR analysis and is capable of accomplishing seven modeling steps: (1) structure preprocessing, (2) molecular descriptor calculation, (3) data preprocessing, (4) feature selection, (5) model building and prediction, (6) Model interpretation (7) statistical analysis. These seven steps together with several ancillary tools are implemented in six modules: (1) *User space*, (2) *Structure preprocessing*, (3) *Data preprocessing*, (4) *Modeling process*, (5) *Model interpretation* (6) *Tools*. The six modules form an integrated pipeline for modeling, but each of these modules can also be used as a standalone tool. The whole workflow is shown in Fig. 1.

## Methods

### Implementation

**Fig. 2:**
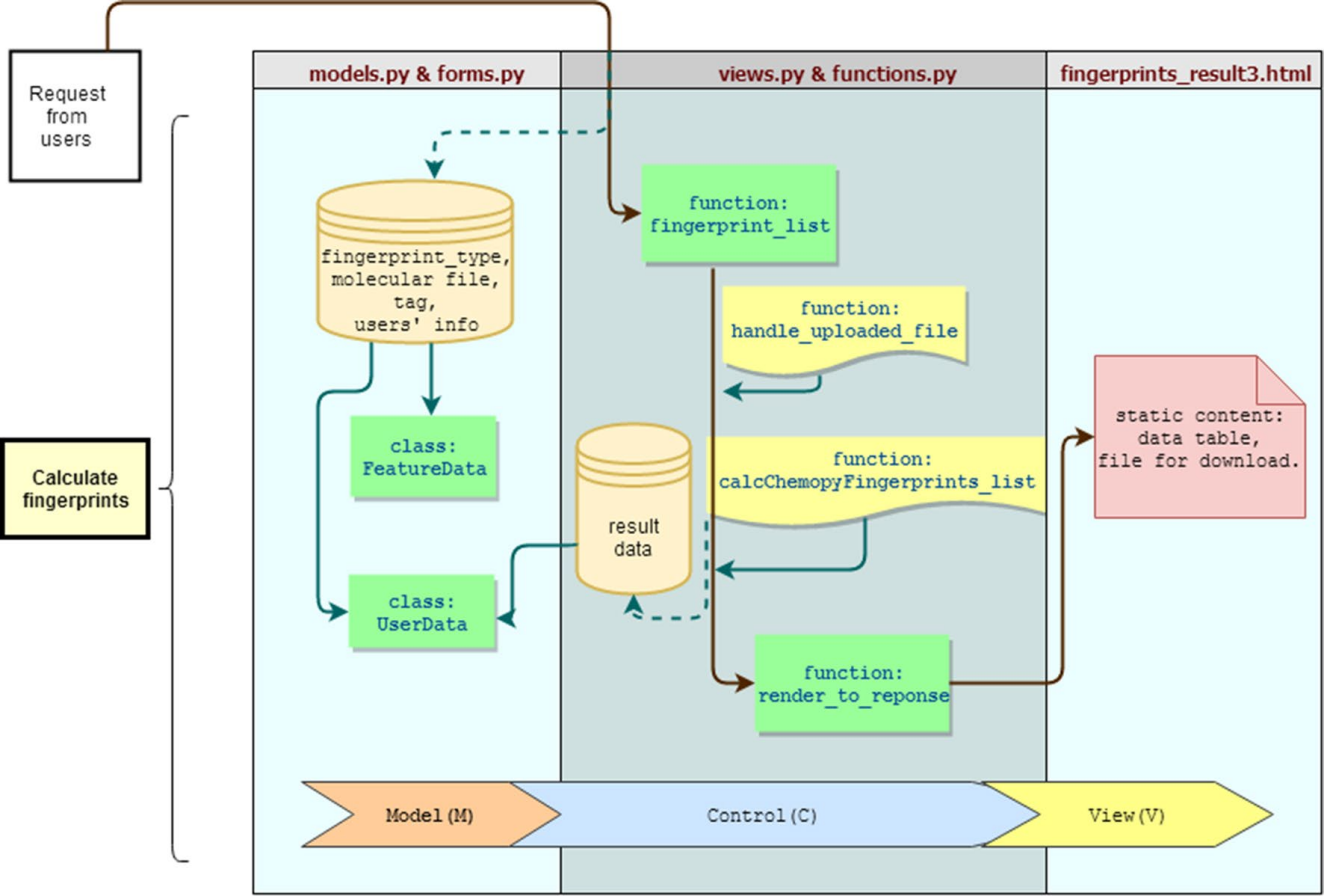
The process of calculating molecular fingerprints—an example to explain the development methods of ChemSAR

The whole project runs on an elastic compute service (ECS) server of Aliyun. The number of CPU cores and memory are automatically allocated to the running instances on demand, which ensures the elastically stretchable computing capability. Python/Django and MySQL are used for server-side programming, and HTML, CSS, JavaScript are employed for front side web interfaces. The realization of functionality should go through three main components (MCV short for Model-Control-View model). To illustrate the implementation of the ChemSAR architecture, we consider the functionality of “Feature Calculation” as an example and the corresponding diagram is shown in Fig. 2 (see also Additional file 1). This module consists of four *.py files and a template folder: *models.py* acts as “M” to access the database; *views.py* acts as “C” to realize the functionality; the *functions.py* acts as a library to store the key calculation procedures which could be called into *views.py*; the *forms.py* stores input forms that can be used in templates; The HTML pages in the template folder act as “V” to visualize the results. Firstly, users go to the index page of “Feature Calculation” and submit the request. Then, the function: *fingerprint_list* (from *views.py*) executes as the back-end calculation program. In this function, (1) the input data from users is handled; (2) the input data is saved into database; (3) the key calculation function is called and executed; (4) the calculation result is stored into database and provided as file for download; (5) all the related variables are rendered into content for view. Among them, *UserData* and *FeatureData* are called from *models.py* to store users’ data and result data; *handle_uploaded_file* and *calcChemopyFingerprints_list* are imported from *functions.py* to store the uploaded file and calculate specified fingerprints. At last, the calculated fingerprints values will be rendered to static contents displayed in *fingerprint_result3.html*. As an easy-to-use web service, ChemSAR supports commonly-used file formats for data exchange between the server-side and the client-side. Specifically, simplified molecular input line entry specification (SMILES) and Structure Data Format (SDF) are acceptable molecular file formats (or users can convert their files into these two formats using OpenBable [37] or ChemCONV [49]). The modeling results will be presented as HTML web pages, but users can download the results in SDF, CSV, PNG or PDF format (see Table 1 for details).

**Table 1:** The valid file formats and requirements for each module

Module name	Input	Output	Description
Feature calculation	*.smi; *.sdf	*.csv	The standard version of molfile in SDF must be V2000; 2D or 3D information are both valid; the first row of *.csv file is the descriptor names; the first column is the SMILES of molecules
Model selection	*.csv	Data table in the page	The first column of X_train file can be molecular identifiers like molecular names or IDs; the first row of X_train file must be descriptor names; the first column of y_train file must be the same with X_train file; the second column must be experimental values of the sample (different presentation styles of classes must be converted into 0 or 1)
Model building	*.csv	Data table in the page; *.png	The same with “Model selection”
Prediction	*.csv	Data table in the page; *.csv	The requirements of X_test file are the same with X_train file
Validation of molecules	*.sdf	*.csv	The standard version of molfile in SDF must be V2000
Standardization of molecules	*.sdf	*.sdf	The same with “Validation of molecules”
Custom preprocessing	*.sdf	*.sdf	The same with “Validation of molecules”
Imputation of missing values	*.csv	*.csv	The first row of input file must be header like descriptor names; each column including the first one must be feature values like descriptor values
Removing low variance features	*.csv	*.csv	The same with “Imputation of missing values”
Removing high correlation features	*.csv	*.csv	The same with “Imputation of missing values”
Univariate feature selection	*.csv	*.csv	The first row of input file must be header like descriptor names; each column from the first one to the penultimate one must be feature values like descriptor values; The last column must be experimental values of the sample (different presentation styles of classes must be converted into 0 or 1)
Tree-based feature selection	*.csv	Data table in the page; *.csv	The same with “Univariate feature selection”
RFE feature selection	*.csv	Data table in the page; *.csv; *.png	The same with “Univariate feature selection”
Statistical analysis	*.csv	Data table in the page; *.png	The four columns of input file must be in order: molecular identifier, predict label, predict probability, experimental value; the label name can be defined by users
Random training set split	*.csv	*.csv	The same with “Model selection”
Diverse training set split	*.sdf	*.sdf	The de facto standard version of molfile in SDF must be V2000; 2D or 3D information are both valid
Feature importance	*.csv	*.csv	The same with “Univariate feature selection”

### User interface

To accomplish the complex modelling steps by using a web-based tool, a user-friendly interface is very necessary. In ChemSAR, database and session technologies are utilized to help develop a complete job submission and user space system. The AJAX technology is used in those processes that usually take a long time to finish, which makes it possible to check the status of different jobs at a convenient time. Besides, a logging system is developed to make sure that every step or wrong operation will trigger user-friendly tips or messages. The user interface consists of three main parts: the “Model” section, “Manual” section and “Help” section. The “Model” section is the main entrance for s*tructure preprocessing*, *data preprocessing*, *modelling process* and *tools*. The “Manual” section describes theories and requirements for each module. The “Help” section gives detailed explanations of each module and a standard example of each step of building an SAR classification model.

## Module functionality

### User space

In general, to build a model, there is a need of storage space for user’s data and computing results. In this project, the “*User space*” module is developed to enable users to view, download and reuse all related files or models at any time.

### Structure preprocessing

It is very difficult for SAR practitioners to collect and integrate chemical structures from multiple sources due to the use of different structural representations, diverse file formats, distinct drawing conventions and the existence of labeling mistakes in such sources [66]. Therefore, preprocessing and standardizing these structures are very important tasks that will ensure the correctness of graphical representation and the consistence of molecular property calculation. Hence, we developed the “*Structure preprocessing*” module for molecule structure standardization based on the RDKit package. This module consists of three sub-modules, called *Validation of molecules*, *Standardization of molecules*, and *Custom preprocessing*, respectively. The “*Validation of molecules*” sub-module can check and visualize molecular structures. A warning message will be triggered if any anomaly is detected (e.g., a molecule has zero atoms, or has multiple fragments, or is not an overall neutral system, or contains isotopes), and both the molecular structure and the validation result will be displayed in an interactive table. The “*Standardization of molecules*” sub-module consists of the following steps: removing any hydrogen from the molecule, sanitizing the molecule, breaking certain covalent bonds between metals and organic atoms, correcting functional groups and recombining charges, re-ionizing a molecule such that the strongest acids ionize firstly, discarding tautomeric information and retaining a canonical tautomer. Different from the one-click process implemented in “*Standardization of molecules*”, the “*Custom preprocessing*” sub-module provides flexible options for users to construct customized standardization process according to their own preferences of operations and execution orders.

### Data preprocessing

Feature selection is one of the focuses of many SAR-based researches, for which datasets with tens (or hundreds) of thousands of variables need to be analyzed and interpreted. The variables from the descriptor calculation step usually need to be selected for the following reasons: removing unneeded, irrelevant or redundant features, simplifying models for easiness of interpretation, and shortening training time [67]. This module is built upon the *scikit*-*learn* package and consists of six sub-modules, including imputation of missing values (*imputer*), removal of low variance features (*rm_var*), removal of highly correlated features (*rm_corr*), univariate feature selection (*select_univariate*), tree-based feature selection (*select_tree_based*) and recursive feature elimination (*select_RFE*). The *imputer* module can impute missing values (e.g., nan) in the data. The *rm_var* and *rm_corr* modules remove features by a predefined threshold of variance or correlation coefficient without incurring a significant loss of information. The *select_univariate* module works by selecting *k* best features based on univariate statistical tests (e.g., Chi square or F tests). The *select_tree_based* module discards trivial features according to the importance computed using an estimator of randomized decision trees. The *select_RFE* module performs recursive feature elimination in a cross-validation loop to find the optimal number of features. Here, an estimator of support vector classification with a linear kernel is invoked to compute a cross-validated score for each recursive calculation. After the calculation, a figure is displayed on the result page to show the relationship between the number of features and the cross-validation scores. A table that contains the optimal number of features, the feature ranking and the cross-validation scores is also presented there.

### Modeling process

The core steps of building an SAR model are implemented in the “*Modeling process*” module. It contains four sub-modules: *feature calculation*, *model selection*, *model building*, and *prediction*.

#### Feature calculation

In this project, we developed the *feature calculation* sub-module as an online tool [36], which allows users to calculate 783 molecular descriptors from 12 feature groups (see Table 2). These features cover a relatively broad range of molecular properties and are carefully selected based on our experience. In recent years, molecular fingerprints are widely used in drug discovery area, especially for similarity search, virtual screening and QSAR/SAR analysis due to their computational efficiency when handling and comparing chemical structures. In this sub-module, ten types of molecular fingerprint algorithms are implemented (see Table 2). These molecular fingerprints have been shown to have a good performance in characterizing molecular structures.

**Table 2:** The list of molecular descriptors computed by ChemSAR

Feature group	Features	Number of descriptors
Constitution	Molecular constitutional descriptors	30
Topology	Topological descriptors	35
Connectivity	Molecular connectivity indices	44
E-state	E-state descriptors	245
Kappa	Kappa shape descriptors	7
Basak	Basak descriptors	21
Burden	Burden descriptors	64
Autocorrelation	Moreau-Broto autocorrelation	32
	Moran autocorrelation	32
	Geary autocorrelation	32
Charge	Charge descriptors	25
Property	Molecular property	6
MOE-type	MOE-type descriptors	60
CATS	CATS descriptors	150
Fingerprints	Topological-Torsion fingerprints	1024
	MACCS keys	167
	FP4 fingerprints	307
	FP2 fingerprints	1024
	FP3 fingerprints	210
	E-state fingerprints	79
	Daylight-type fingerprints	1024
	ECFP2 fingerprints	1024
	ECFP4 fingerprints	1024
	ECFP6 fingerprints	1024

#### Model selection

The *model selection* sub-module is developed to select a proper learning algorithm and computing parameter set based on user’s dataset via comparing/validating models and tuning parameters. Five learning algorithms [68–72] from the *scikit*-*learn* package are implemented. These algorithms, along with their parameters and the recommended defaults, are listed in Table 3. The detailed description of each algorithm and how to choose proper algorithms for different datasets are described in the “Manual” section of the website. After the calculation, a table is created to display the *classifier*, *parameter*, *best parameter*, *number of positive samples*, *number of negative samples* and *score_means* (the mean score of cross-validation for each parameter combination).

**Table 3:** The supported algorithms and related parameters

Algorithms	Parameters	Recommended parameters
RandomForest	*n_estimators*: The number of trees in the forest; *max_features*: The number of features to consider when looking for the best split; (*start_feature*, *end_feature* and step make up the attempts of *max_features*) *cv*: cross-validation fold	*n_estimators*:500; *max_features*: sqrt(N); N stands for number of features; *cv*: 5
SVM	*kernel type*: rbf, sigmoid, poly, linear; *C*: penalty parameter C of the error term.; *gamma*: kernel coefficient for ‘rbf’, ‘poly’ and ‘sigmoid’; *degree*: degree of the polynomial kernel function; *cv*: cross validation fold	*C*: 2^−5, 2^15, 2^2; (format: start, end, step) *gamma*: 2^(−15), 2^3, 2^2; *degree*: 1, 7, 2 *cv*: 5
Naïve Bayes	*Bayes classifier type*; *cv*: cross validation fold	*BernoulliNB* for binary-valued variable; *GaussianNB* for continuous variable; *cv*: 5
K Neighbors	*n_neighbors*: number of neighbors to use; *cv*: cross validation fold	*n_neighbors*: 1–10; *cv*: 5
DecisionTree	*Algorithm*: algorithm used to compute the nearest neighbors (‘ball_tree’, ‘kd_tree’, ‘brute’); *cv*: cross validation fold	Automatic decision; *cv*: 5

#### Model building

In the previous sub-module, a proper learning algorithm and the corresponding parameter set have been obtained. In this sub-module, a predictive classification model based on the selected method and parameters can be established. Here the same training dataset as that in the *model selection* sub-module should be chosen (or uploaded manually). The result table generated from this sub-module will give detailed information about the model, including *classifier*, *parameter*, *number of positive samples*, *number of negative samples*, *AUC score*, *accuracy*, *MCC* (Matthews correlation coefficient), *F1 score*, *sensitivity*, *specificity* and *ROC curve*. The built model will be stored in *my model* and can be employed to predict new samples next time.

#### Prediction

This sub-module is used to predict the samples from the test set or new samples from virtual chemical libraries, which is the ultimate goal of building an SAR model. In the submitting page, users can check the detailed information about the model including the *classifier*, *parameter*, *accuracy*, *cross*-*validation fold*, *features in* **X** (i.e., selected features used in training set), **X** *train*, **y** *train*. When the file of the test set matrix is uploaded, the model will calculate the *predict_prob* and *predict_label* for submitted samples. In the result page, an interactive table containing prediction results will be displayed and can also be downloaded.

### Model interpretation and application domain

It is very necessary to have a reasonable interpretation of machine learning models and to define its application domain [66, 73, 74]. Here, we developed two related sub-modules to help researchers to interpret their models. The *feature importance* module enables researchers to interpret models in terms of feature importance. The forests of trees are used to evaluate the importance of features. By using this module, researchers can obtain a figure displaying the feature importances of the forest, along with their inter-trees variability. Another module is *tree visualization* which enables one to observe how the features classify the samples step by step in the decision tree model. By using this, the model will be displayed as a clear tree along with class names and explicit variables.

Moreover, we define an *S index* in the *prediction* module to help users to estimate which ones are considered reliable. It only works for chemical datasets. The *S indices* represent the mean similarity between each molecule from external samples and all molecules from training set using Tanimoto similarity metrics based on MACCS fingerprints. The higher the *S index* for a new molecule, the closer the molecule is to the main body of the training set, and thus we could conclude that a more reliable prediction for this molecule should be obtained by our constructed predictive model.

### Report system

One of the striking features of ChemSAR is that it provides a complete report generation system. It retrieves the results of each calculation step and re-arrange them into an organized HTML page and a PDF file for users. After finishing the whole modelling pipeline, the user can go to the “*My Report*” module to obtain the report. At the index page of this module, all job IDs that the user has created will be listed there. A “Get a PDF” button allows the user to generate a PDF file for off-line usage. A “Query” button is available to query the information about models created in other jobs. This is very helpful when a user attempts to construct multiple models using different machine learning methods and computing parameters, or when the user wants to build more than one model by the same client at the same time.

### Useful tools

In addition to main modules mentioned above, ChemSAR offers three useful and convenient auxiliary tools. The first tool, *statistical analysis*, can be used to analyze the model performance. This tool is separate from the *prediction* module because the test set may have no real response values. The “attach to current job or not” option allows the user to predict different test sets and get a complete report each time. After the calculation, commonly used statistical indicators related to classification are displayed, including *number of positive samples*, *number of negative samples*, *AUC score*, *accuracy*, *MCC*, *F1 score*, *sensitivity*, *specificity* and *ROC curve*. The second tool, *random training set split*, can be used to split training set and test set by picking a subset of molecules randomly. The third tool, *diverse training set split*, can be used to split training set and test set by picking a subset of diverse molecules [75]. First, the similarity of ECFP4 fingerprints based on Dice similarity metric [31] is employed to calculate distances between molecular objects and then the MinMax algorithm is applied to select a subset of diverse molecules based on the aforementioned distances. This is usually a good strategy to avoid the unsuitable training/test data split problem.

## Results and discussion

**Fig. 3:**
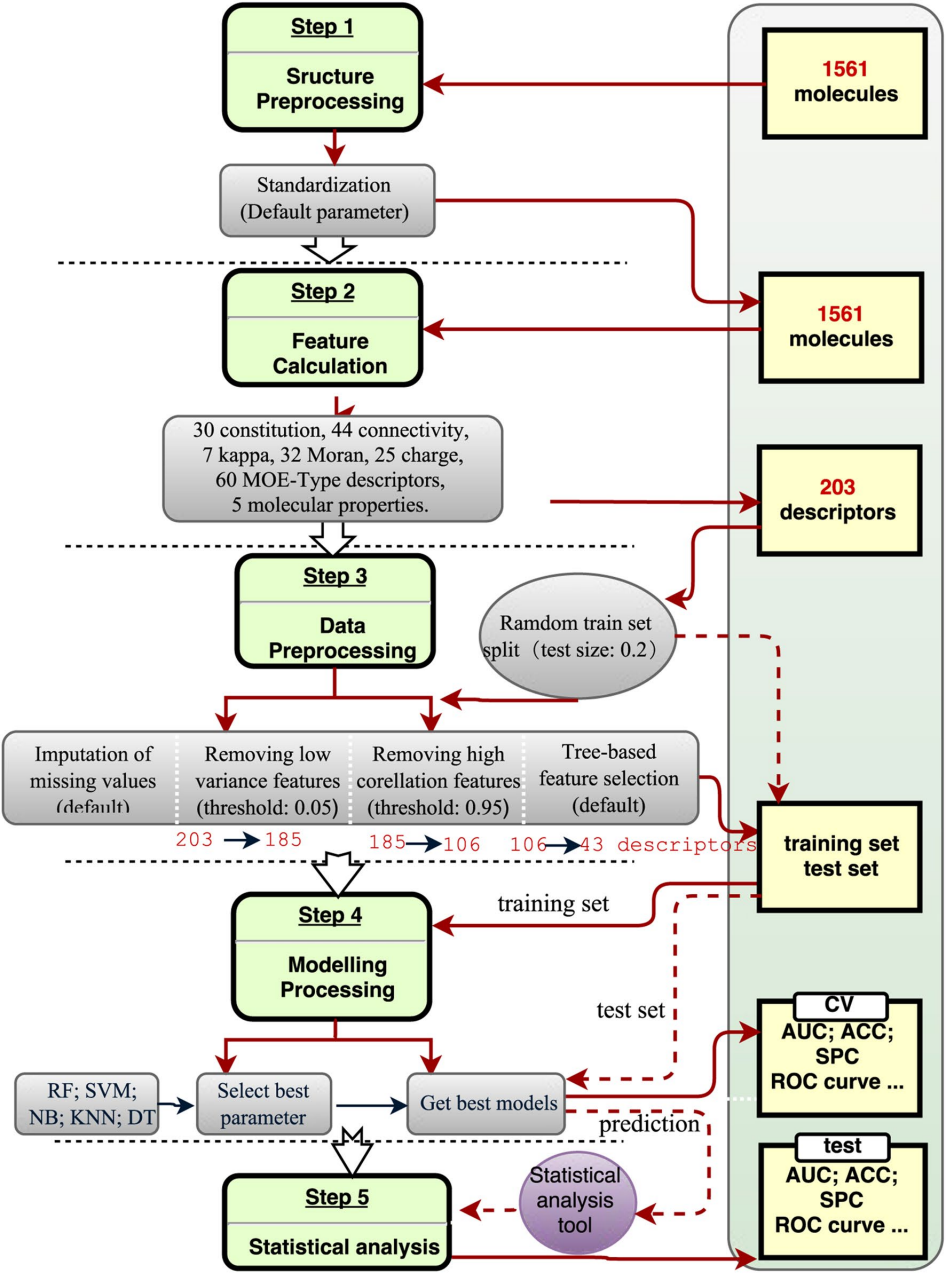
The workflow of building Caco-2 Cell permeability models

The most important strategy of pharmaceutical industry to overcome its productivity crisis in drug discovery is to focus on the molecular properties of absorption, distribution, metabolism and excretion (ADME). Nowadays, machine learning based approaches have been becoming a very popular choice to predict ADME properties of drug molecules. Here, in order to demonstrate the practicability and reliability of ChemSAR, we studied the Caco-2 Cell permeability using dataset from our previous publication [12] . All the compounds were divided into two classes according to the Caco-2 permeability cutoff value [12]. Then, we obtain a dataset of 1561 molecules containing 528 positive samples and 1033 negative samples. A detailed workflow of building the permeability models is shown in Fig. 3.

Firstly, through the structure preprocessing step (*Standardization of molecules*) using the default parameters, 1561 molecules were left. The *random training set split* tool (set test size for the data: 0.2) was then used to split the training set and test set. After this, a training set of 1249 samples (423 positive samples and 826 negative samples) and a test set of 312 samples (105 positive samples and 207 negative samples) were obtained.

In the feature calculation step, 203 descriptor were calculated including 30 constitution descriptors, 44 connectivity indices, 7 kappa indices, 32 Moran auto-correction descriptors, 5 molecular properties, 25 charge descriptors and 60 MOE-Type descriptors. Four filtering steps in data preprocessing were then performed: (1) missing values were imputed with the default parameters, and (2) descriptors with zero variance or near zero variance were removed with a cut-off value of 0.05, and (3) one of two highly correlated descriptors were randomly removed with a cut-off value of 0.95, and (4) perform the tree-based feature selection (n_estimators: 500, max_features: auto, threshold: mean). After these steps, 43 features were selected to build the model.

To test every module of ChemSAR and to build a model with high prediction performance, we employed five methods (RF, SVM, *k*-NN, NB, DT) to build classification models. In model selection, the parameters for each learning method were optimized using grid search strategy (The best parameters set for RF: {‘cv’: 5, ‘max_features’: 9, ‘n_estimators’: 500}; SVM: {‘kernel’: ‘rbf’, ‘C’: 2, ‘gamma’: 0.125}; *k*-NN: {n_neighbors: 5}; NB: {‘classifier’: ‘GaussianNB’}.) Then, a robust model was established with a 5-fold cross validation again. Each of the modelling processes will be repeated 10 times and then the statistical results will be reported as “mean ± variance”. Additionally, to test the fingerprints module and to make a further comparison, we also calculated five kinds of fingerprints and then built corresponding models. The model performance was displayed in Additional file 2: Fig. S1 and Additional file 2: Table S1. From the results, we can find that RF using 2D descriptors performs best: {Accuracy: 87.3±0.3, Sensitivity: 80±0.6, Specificity: 91.1 ± 0.3, MCC: 71.5 ± 0.6, AUC: 92.9 ± 0.3} for training set and {Accuracy: 85.3, Sensitivity: 77.1, Specificity: 89.4, MCC: 66.8, AUC: 89.9} for test set. Consequently, the RF method is more suitable for building a classification model for this dataset and the 2D descriptors can characterize molecules of this dataset more adequately. Clearly, by using the modelling module in ChemSAR, one could conveniently construct different algorithm models for one dataset and then makes a comprehensive comparison and further analysis to identify the best prediction model for the current problem.

To further evaluate the prediction ability of our models, we compared our prediction results with the published models in recent papers. The latest report was in 2013 [76], the authors built a model using DT method with 1289 compounds which could accurately predict 78.4/76.1/79.1% of H/M/L compounds on the training set and 78.6/71.1/77.6% on the test set. In 2011 [77], Pham et al. built a model using linear discriminant analysis (LDA) method with 674 molecules which reported results: MCC = 0.62, Accuracy = 81.56% (training set), Accuracy = 83.94% (test set). Compared with the two models above, our model has an almost comparative or better performance. Obviously, ChemSAR has the capacity to obtain the reliable and robust classification model for the evaluation of Caco-2 Cell permeability.

### Comparison with other related tool sources

For the purpose of further comparison, we have studied related publications as much as we can, and searched on Google to collect related tools that possess SAR modelling functionality. Then, a comparison based on application scenarios and functionality was performed, which was summarized in Table 4. In this table, we compared and then marked several aspects of each tool, including “type”, “structure preprocessing”, “data preprocessing”, “molecular representation”, “feature selection”, “model selection”, “algorithm type”, “charge or free” and “coupling”. The results suggest that ChemSAR is strongly recommended for multiple advantages of it as shown in the table. Note that we cannot absolutely guarantee the accuracy of the description for each tool because we get all the available information mainly from the corresponding publications or its documentations but some tools are not accessible or commercial. Also, the features of each tool evaluated here are from this tool’s main framework, not the plugins provided by its user community.

**Table 4:** The current tools that can be used for SAR modelling

Tool name	Type	Structure preprocessing	Data preprocessing	Molecular representation	Feature selection	Model selection	Algorithm type	Fees/register	Coupling
ChemSAR	Online	✓	✓	✓	✓	✓	I	Free	Low
OCHEM	Database	✓	✓	✓		✓	I, II	Restricted	High
Chembench	Online	✓	✓	✓	✓	✓	I, II	Need register	High
Vcclab	Online				✓	✓	I	Free	Low
OpenTox	Online						For toxicity prediction	Free	High
QSAR4U	Online			✓			Built-in models	Free	High
camb	R package	✓	✓	✓	✓	✓	I, II	Free	Low
AZOrange	Software			✓	✓	✓	Disabled for invalid dependences	Free	Low
RRegrs	R package				✓	✓	II	Free	Low
eTOXlab	Software	✓	✓	✓	✓		Models for production environments	Free	High
QSARINS	Software		✓	✓			II	Restricted	High
OECD QSAR Toolbox	Software			✓			Models for data gap filling	Restricted	High
MOLGEN QSPR	Software			✓			II	Free	High
BuildQSAR	Software				✓		II	Free	High
McQSAR	Software				✓		II	Free	High
StarDrop	Software			✓		✓	I, II	Commercial	–
MOE	Software	✓		✓			I, II	Commercial	Low
DS	Software		✓	✓			I, II	Commercial	Low

The I and II represent the classification algorithms and regression algorithms; The “restricted” means that some modules of the tool are limited to the public or need the permits of the developers; The “low” coupling means that the main modules of the tool can be called in the modelling pipelining and can also be used as an independent tool, while the “high” coupling means that they must work together to build a model

## Conclusion

In this study, we developed the ChemSAR platform as an online pipelining of building SAR classification models. It is freely accessible to the public and is platform-independent so users can access this platform via almost all different types of operation systems (Linux, Microsoft windows, Mac OS, Android) and clients (PC clients, mobile clients). The main advantages of the proposed platform are summarized as follows: (1) ChemSAR implements a complete online model-building process, which enables biomedical investigators to construct predictive models easily without suffering from tedious programming and deployment work. (2) ChemSAR provides a comprehensive modelling pipelining by integrating six model generation steps into a unified workflow. (3) The modular design of the framework enables six sub-modules to run independently to accomplish specific functionalities. (4) The job submission strategy allows users to query the calculation results at spare time. This provides an essential basis for the report system to generate a clear modeling report. (5) The modular design of the framework allows researchers to deal with not only the analysis of small molecules but also modelling problems in the biomedical field. For example, building a classification model based on the biochemical indicators of patients helps to study the disease classification or stage. In addition, we conducted a case study to illustrate the use of this platform in practice, and several models were obtained to evaluate the Caco-2 Cell permeability at the same time.

A major goal of the cheminformatics development is to make its techniques to be applied into the study of practical problems. The trend for future development of SAR models is towards making models publicly accessible on-line, interactive, and usable [78]. ChemSAR, to some extent, has made a step in this direction. It is expected that ChemSAR can be applied to a wide variety of studies when there exists a significant demand of using SAR models. In the future, we will continue to implement more classification algorithms and add the options for a more flexible parameter control. Also, we will add the regression algorithms if needed.

## Additional files

**Additional file 1:** The code snippets to show the implementation of calculating molecular fingerprints.

**Additional file 2: Table S1.** Classification results of different models in the evaluation of Caco-2 Cell permeability. **Fig. S1.** The ROC curves for different models in the evaluation of Caco-2 Cell permeability.
