# Supplementary figures and images for: ChemSAR: an online pipelining platform for molecular SAR modeling
Source: J Cheminform. 2017 May 4;9:27. doi: 10.1186/s13321-017-0215-1 (PMC5418185; doi:10.1186/s13321-017-0215-1)

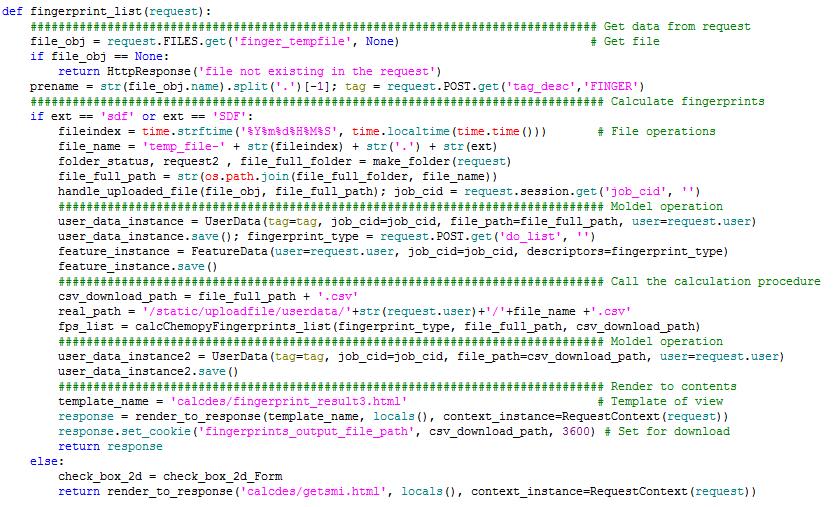

Supplement: Supplementary file 1 — Additional file 1: The code snippets to show the implementation of calculating molecular fingerprints. [file 13321_2017_215_MOESM1_ESM.zip › code_ snippet_1.jpg]

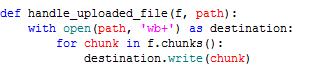

Supplement: Supplementary file 1 — Additional file 1: The code snippets to show the implementation of calculating molecular fingerprints. [file 13321_2017_215_MOESM1_ESM.zip › code_ snippet_2.jpg]

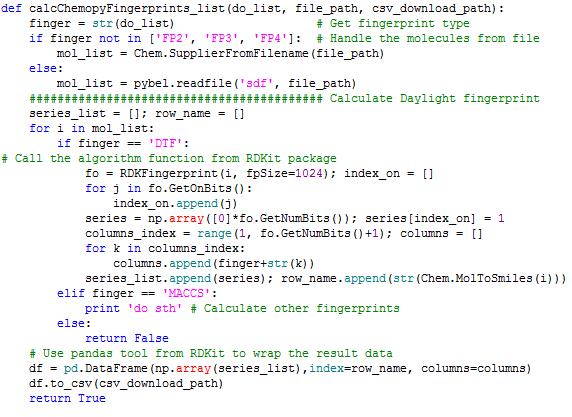

Supplement: Supplementary file 1 — Additional file 1: The code snippets to show the implementation of calculating molecular fingerprints. [file 13321_2017_215_MOESM1_ESM.zip › code_ snippet_3.jpg]

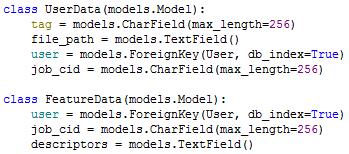

Supplement: Supplementary file 1 — Additional file 1: The code snippets to show the implementation of calculating molecular fingerprints. [file 13321_2017_215_MOESM1_ESM.zip › code_ snippet_4.jpg]
