# Supplementary material for: ChemSAR: an online pipelining platform for molecular SAR modeling
Source: J Cheminform. 2017 May 4;9:27. doi: 10.1186/s13321-017-0215-1 (PMC5418185; doi:10.1186/s13321-017-0215-1)
Supplement: Supplementary file 2 — Additional file 2: Table S1. Classification results of different models in the evaluation of Caco-2 Cell permeability. Fig. S1. The ROC curves for different models in the evaluation of Caco-2 Cell permeability. [file 13321_2017_215_MOESM2_ESM.docx]

| **Table S1** Classification results of different models in the evaluation of Caco-2 Cell permeability | | | | | | | | | | | |
| --- | --- | --- | --- | --- | --- | --- | --- | --- | --- | --- | --- |
| Method | Feature type | 5-fold cross validation | | | | | External test | | | | |
|  |  | Accuracy | Sensitivity | Specificity | MCC | AUC | Accuracy | Sensitivity | Specificity | MCC | AUC |
| RF | **2D descriptors** | **87.3±0.3** | **80.0±0.6** | **91.1±0.3** | **71.5±0.6** | **92.9±0.3** | **85.3** | **77.1** | **89.4** | **66.8** | **89.9** |
|  | Daylight-type | 84.5±0.5 | 68.4±1.2 | 92.8±0.9 | 64.5±1.3 | 90.1±0.5 | 82.7 | 63.8 | 92.3 | 60.0 | 87.7 |
|  | ECFP4 | 86.1±0.6 | 72.7±1.3 | 92.9±0.4 | 68.3±1.4 | 91.9±0.4 | 81.4 | 64.8 | 89.9 | 57.2 | 88.3 |
|  | E-state | 85.7±0.6 | 75.3±1.2 | 91.1±0.6 | 67.7±1.4 | 91.5±0.5 | 81.4 | 69.5 | 87.4 | 57.8 | 86.8 |
|  | FP4 | 83.8±0.6 | 73.4±1.5 | 89.1±0.4 | 63.4±1.4 | 89.5±0.4 | 79.5 | 61.0 | 88.9 | 52.5 | 85.6 |
|  | MACCS | 84.4±1.1 | 68.4±1.9 | 92.6±0.8 | 64.3±2.5 | 90.0±0.8 | 82.7 | 63.8 | 92.3 | 60.0 | 87.6 |
| SVM | **2D descriptors** | **86.5±0.4** | **74.1±1.1** | **92.8±0.4** | **69.2±0.9** | **90.5±0.6** | **80.4** | **79.0** | **61.4** | **54.7** | **85.3** |
|  | Daylight-type | 83.2±0.5 | 66.0±1.4 | 92.0±0.4 | 61.4±1.2 | 88.2±0.6 | 82.1 | 59.0 | 93.7 | 58.4 | 84.6 |
|  | ECFP4 | 85.5±0.4 | 76.4±0.8 | 90.1±0.5 | 67.3±0.9 | 91.0±0.4 | 79.8 | 65.7 | 87.0 | 53.9 | 85.1 |
|  | E-state | 84.0±0.6 | 75.6±1.0 | 88.3±0.8 | 64.2±1.2 | 89.4±0.4 | 81.7 | 67.6 | 88.9 | 58.2 | 85.3 |
|  | FP4 | 83.7±0.6 | 75.0±0.8 | 88.2±0.8 | 63.5±1.2 | 87.9±0.5 | 81.7 | 67.6 | 88.9 | 58.2 | 84.6 |
|  | MACCS | 83.4±0.5 | 65.6±1.4 | 92.4±0.4 | 61.7±1.1 | 88.2±0.6 | 82.1 | 59.0 | 93.7 | 58.4 | 84.6 |
| NB | 2D descriptors | 70.1±0.2 | 83.8±0.4 | 63.1±0.4 | 44.5±0.4 | 78.4±0.1 | 67.3 | 79.0 | 61.4 | 38.2 | 74.5 |
|  | Daylight-type | 62.0±0.2 | 56.3±0.4 | 64.9±0.2 | 20.3±0.4 | 62.0±0.1 | 60.3 | 49.5 | 65.7 | 14.7 | 57.6 |
|  | **ECFP4** | **77.5±0.8** | **76.4±1.4** | **78.0±0.5** | **52.5±1.7** | **85.6±0.4** | **72.4** | **73.3** | **72.0** | **43.2** | **82.2** |
|  | E-state | 71.8±0.6 | 56.0±1.0 | 79.8±0.7 | 36.3±1.4 | 75.9±0.2 | 67.3 | 52.4 | 74.9 | 27.1 | 70.5 |
|  | FP4 | 74.0±0.3 | 70.2±0.7 | 75.9±0.6 | 44.6±0.6 | 81.0±0.3 | 71.8 | 68.6 | 73.4 | 40.4 | 77.9 |
|  | MACCS | 61.9±0.1 | 56.3±0.3 | 64.8±0.2 | 20.2±0.3 | 62.0±0.2 | 60.3 | 49.5 | 65.7 | 14.7 | 57.6 |
| KNN | 2D descriptors | 82.3±0.6 | 74.1±1.4 | 86.5±0.5 | 60.5±1.5 | 87.7±0.6 | 78.2 | 68.6 | 83.1 | 51.4 | 84.9 |
|  | **Daylight-type** | **83.4±0.7** | **75.2±1.5** | **87.6±0.6** | **62.9±1.6** | **81.4±0.9** | **82.4** | **68.6** | **89.4** | **59.7** | **79.0** |
|  | ECFP4 | 78.9±0.9 | 82.1±1.0 | 77.2±0.9 | 56.7±1.7 | 79.7±0.9 | 76.0 | 75.2 | 76.3 | 49.5 | 75.8 |
|  | E-state | 82.8±1.0 | 76.4±1.9 | 86.0±0.9 | 61.9±2.2 | 81.2±1.2 | 77.6 | 61.9 | 85.5 | 48.7 | 73.7 |
|  | FP4 | 80.0±1.0 | 74.7±1.7 | 82.6±0.9 | 56.3±2.2 | 78.7±1.1 | 78.8 | 70.5 | 83.1 | 53.1 | 76.8 |
|  | MACCS | 83.3±0.5 | 75.4±1.6 | 87.3±0.8 | 62.7±1.1 | 81.4±0.6 | 82.7 | 68.6 | 89.9 | 60.4 | 79.2 |
| DT | **2D descriptors** | **86.5±0.4** | **74.1±1.1** | **92.8±0.4** | **69.2±0.9** | **90.5±0.6** | **82.7** | **77.1** | **85.5** | **61.8** | **82.8** |
|  | Daylight-type | 79.7±1.3 | 70.9±2.1 | 84.2±1.3 | 54.8±2.9 | 78.4±1.5 | 79.2 | 76.2 | 80.7 | 55.2 | 79.0 |
|  | ECFP4 | 79.8±0.9 | 71.8±1.5 | 83.8±0.9 | 55.2±1.9 | 78.4±1.0 | 79.2 | 63.8 | 87.0 | 52.3 | 75.9 |
|  | E-state | 82.1±0.7 | 73.7±2.1 | 86.5±0.8 | 60.1±1.7 | 81.4±0.8 | 76.9 | 64.8 | 83.1 | 48.1 | 75.4 |
|  | FP4 | 80.1±1.0 | 70.7±1.3 | 85.0±1.1 | 55.6±2.1 | 80.8±0.9 | 76.3 | 59.0 | 85.0 | 45.5 | 73.4 |
|  | MACCS | 79.8±0.8 | 70.9±1.7 | 84.4±1.1 | 55.2±1.8 | 78.4±1.0 | 79.5 | 75.2 | 81.6 | 55.5 | 79.5 |
| The bold lines represent the comparative best model of each group. The values are shown in the form of percentage. | | | | | | | | | | | |


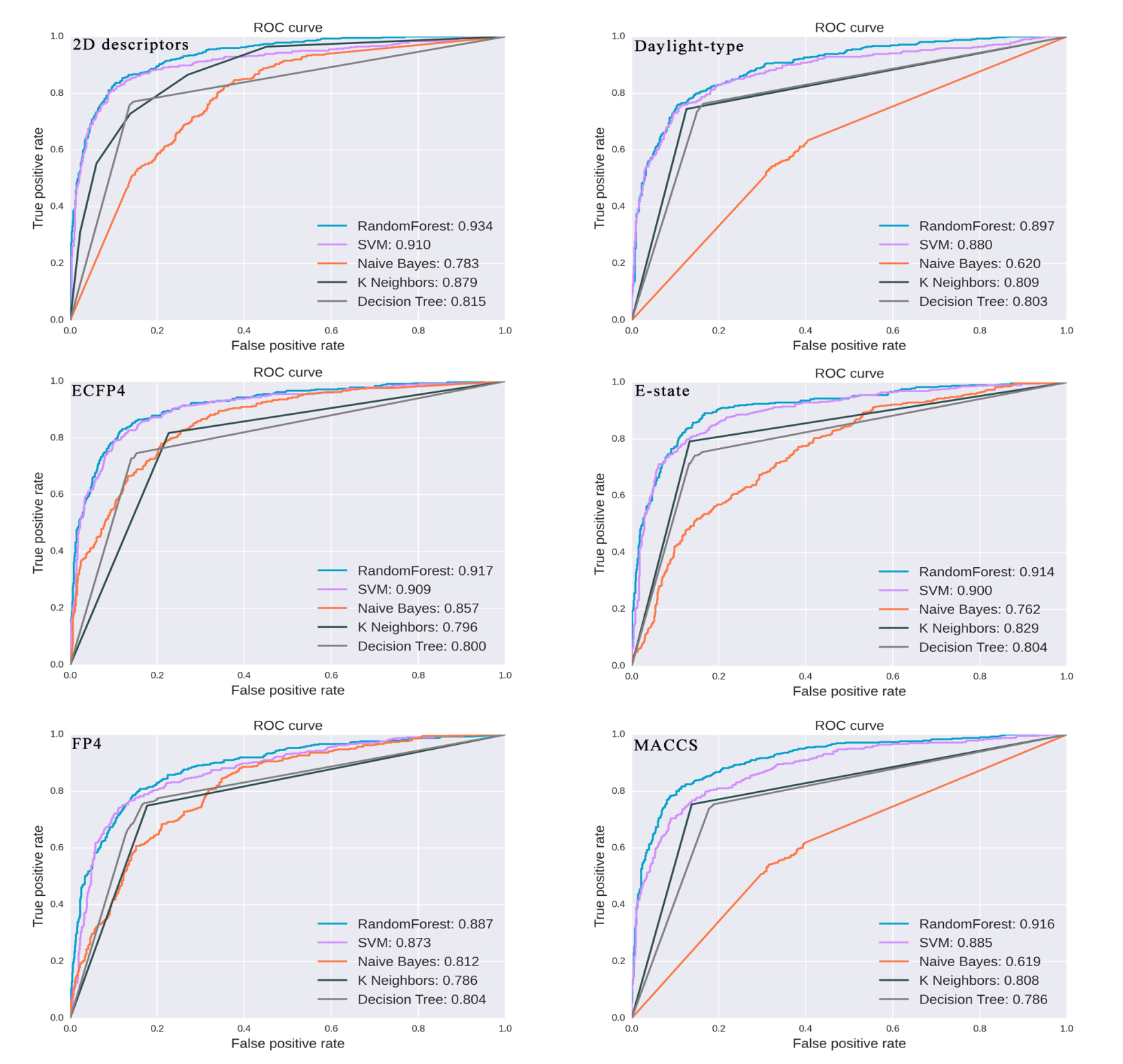
Fig.S1 The ROC curves for different models in the evaluation of Caco-2 Cell permeability
